# JCEM Case Reports: An Opportunity Not to Be Missed

**DOI:** 10.1210/jcemcr/luae155

**Published:** 2024-08-23

**Authors:** William F Young

**Affiliations:** Editor-in-Chief, JCEM Case Reports

At the June 2024 Annual Endocrine Society Meeting in Boston (ENDO2024), we celebrated our first 18 months of publication by hosting our second annual symposium entitled “Clinical Pearls from *JCEM Case Reports*.” Three authors presented their cases and addressed questions from the audience. Following each presentation, an experienced content expert shared their clinical pearls. A recording of this session is available on the journal website (https://academic.oup.com/jcemcr).

At ENDO2024, I had the opportunity to visit with many medical students, internal medicine residents, endocrine trainees, and junior faculty at their case-report posters. I could not visit them all, but I did read all of the case-report abstracts. Many described common endocrine disorders with unique diagnostic, ethical, or management challenges. Other case-report abstracts documented rare endocrine disorders that presented with a new association, unexpected findings, or unanticipated treatment responses. I encouraged the poster presenters to consider submitting their case reports for publication in *JCEM Case Reports*. Although presenting the case reports at ENDO2024 was a valuable experience for the presenters and those who visit the posters, the impact of sharing their case-based key learning points is increased more than 10 000-fold when published in *JCEM Case Reports*.

Many of my best teaching and learning opportunities have been based on the care of individual patients with perplexing clinical scenarios. When I was an internal medicine resident, my very first clinical publication was a case report describing a patient with a pituitary gland disorder that was a diagnostic conundrum [1]. Caring for that patient and writing the report helped ignite my interest in pursuing a career in endocrinology. Too often, the clinical pearls that we learn from managing difficult clinical situations are never shared. *JCEM Case Reports* is the forum for endocrinologists (and those aspiring to be endocrinologists!) to pass on these valuable clinical insights.

Recognizing that many manuscripts are submitted by clinicians in training, our editorial team is charged to evaluate the methodologic quality of case reports and to coach the authors on how to optimize their submissions with respect to case selection, ascertainment, causality, and reporting. All authors are required to use a uniform manuscript template that includes tips on how to polish their manuscript. The manuscript template and a video on how to use it can be found on the journal website (https://academic.oup.com/jcemcr). Manuscript sections include an introduction, case presentation, diagnostic assessment, treatment, outcome and follow-up, discussion, and bulleted learning points. The optimal case report is factual, concise, well organized, presented clearly, and easily readable. After an accepted manuscript has been copyedited and the final version has been approved, it will appear online in an issue. All content is free for reading worldwide.

For those of you who attended the annual Endocrine Society meeting, I hope you enjoyed it as much as I did. And for those of you who presented a case-report poster, I encourage you to give your case the permanency and exclamation mark that it deserves by submitting it to *JCEM Case Reports*!
